# Effects of Collagen Hydrolysate From Large Hybrid Sturgeon on Mitigating Ultraviolet B-Induced Photodamage

**DOI:** 10.3389/fbioe.2022.908033

**Published:** 2022-06-27

**Authors:** Bei Chen, Lei Yu, Jingna Wu, Kun Qiao, Lulu Cui, Haidong Qu, Yongchang Su, Shuilin Cai, Zhiyu Liu, Qin Wang

**Affiliations:** ^1^ Fisheries Research Institute of Fujian, Key Laboratory of Cultivation and High-Value Utilization of Marine Organisms in Fujian Province, Xiamen, China; ^2^ School of Life Sciences, Xiamen University, Xiamen, China; ^3^ Xiamen Medical College, Xiamen, China; ^4^ College of Food Sciences & Technology, Shanghai Ocean University, Shanghai, China; ^5^ College of the Environment and Ecology, Xiamen University, Xiamen, China

**Keywords:** large hybrid sturgeon, collagen hydrolysate, UVB, anti-photoaging, anti-apoptosis activity, antioxidant

## Abstract

Ultraviolet B (UVB) radiation leads to the excessive accumulation of reactive oxygen species (ROS), which subsequently promote inflammation, degradation of the extracellular matrix, and photoaging in skin. Thus antioxidant activity is particularly important when screening for active substances to prevent or repair photodamage. Marine fish-derived bioactive peptides have become a trend in cosmetics and functional food industries owing to their potential dermatological benefits. In this study, 1-diphenyl- 2-pycryl-hydrazyl (DPPH) scavenging activity was selected to optimize the hydrolysis conditions of sturgeon skin collagen peptides with antioxidant activity. The optimal hydrolysis conditions for sturgeon skin collagen hydrolysate (SSCH) were determined by response surface methodology, which comprised an enzyme dosage of flavorzyme at 6,068.4 U/g, temperature of 35.5°C, pH of 7, and hydrolysis time of 6 h. SSCH showed good radical-scavenging capacities with a DPPH scavenging efficiency of 95%. Then, the effect of low-molecular-weight SSCH fraction (SSCH-L) on UVB irradiation-induced photodamage was evaluated in mouse fibroblast L929 cells and zebrafish. SSCH-L reduced intracellular ROS levels and the malondialdehyde content, thereby alleviating the oxidative damage caused by UVB radiation. Moreover SSCH-L inhibited the mRNA expression of genes encoding the pro-inflammatory cytokines *IL-1β*, *IL-6*, *TNF-α*, and *Cox-2*. SSCH-L treatment further increased the collagen Ⅰα1 content and had a significant inhibitory effect on matrix metalloproteinase expression. The phosphorylation level of JNK and the expression of c-Jun protein were significantly reduced by SSCH-L. Additionally, SSCH-L increased the tail fin area at 0.125 and 0.25 mg/ml in a zebrafish UVB radiation model, which highlighted the potential of SSCH-L to repair UVB-irradiated zebrafish skin damage. Peptide sequences of SSCH-L were identified by liquid chromatography-tandem mass spectrometry. Based on the 3D-QSAR modeling prediction, six total peptides were selected to test the UVB-protective activity. Among these peptides, DPFRHY showed good UVB-repair activity, ROS-scavenging activity, DNA damage-protective activity and apoptosis inhibition activity. These results suggested that DPFRHY has potential applications as a natural anti-photodamage material in cosmetic and functional food industries.

## 1 Introduction

Skin plays an important role as the first barrier in resisting external environmental factors (Qiu et al., 2020). Solar UV radiation is a major example of one these factors and consists of UVA and UVB radiation. Exposure to UVB radiation causes various acute or chronic skin disorders, such as dryness, sunburn, photoaging, pigmentation, and photocarcinogenesis (Ichihashi et al., 2003; Manosroi et al., 2011).

Recently, studies have demonstrated that the mechanism of UVB-induced skin damage includes free radical production-mediated oxidative stress, inflammation, degradation of the extracellular matrix, and DNA damage (Martinez et al., 2015; Xiao et al., 2019). According to the previous research, UVB induces excessive ROS generation via the BLT2-Nox1-linked pathways (Ryu et al., 2010; Glady et al., 2018). Accumulation of ROS induces the activation of MAPK pathways, which promote the activation of the transcription factors NF-κB and AP-1, followed by the upregulated transcription of inflammatory mediators such as NO, iNOS, and Cox-2, and proinflammatory cytokines (such as TNF-α and IL-6). The inflammatory factor will further induce collagen degradation by enhancing the expression of the matrix metalloproteinases (MMPs) such as MMP-1, MMP-3, and MMP-9, preventing the expression of procollagen, and promoting apoptosis in dermal fibroblasts (Subedi et al., 2017; Wang et al., 2020). The expression of MMPs leads to degradation of the extracellular matrix by breaking down type I collagen, elastin, and hyaluronan, which in turn causes skin wrinkles, loss of moisture, and disruption of the skin barrier (Mukherjee et al., 2011; Kim et al., 2020). Through the above mechanism study, ROS is considered to be the prominent biomarkers of UVB photodamage in the skin so that downregulation of oxidative stress is generally used as a strategy to alleviate skin damage (Zhang et al., 2019).

The fish processing industry produces many by-products as waste, which includes skin, head, viscera and bones, et al. These by-product wastes contain good amount of protein rich material that are normally processed into low market-value products, such as animal feed, fish meal and fertilizer. For increasing the value of fish, peptides hydrolysed from fish skin are being prepared by several researchers all over the world. These bioactive peptides has become a trend in cosmeceutical industries due to their broad bioactivities, such as antioxidant, anti-inflammatory, and antimicrobial activities, and wound healing and skin regeneration (Venkatesan et al., 2017). A previous study has demonstrated that the peptide LSGYGP, purified from tilapia fish skin gelatin hydrolysate, has a high hydroxyl radical-scavenging activity, which can significantly reduce intracellular ROS generation in UVB-induced mouse embryonic fibroblasts (Ma et al., 2018). GEIGPSGGRGKPGKDGDAGPK and GFSGLDGAKGD peptides were identified by Lu from cod skin gelatin hydrolysates, and were found to exhibit an anti-photoaging effect by inhibiting MMP-1 expression (Lu et al., 2017). Gelatin isolated from salmon skin and its hydrolysate improves the activities of total superoxide dismutase, plasma glutathione peroxidase**,** and catalase to alleviate UV-induced oxidative damage to skin (Chen et al., 2016).

The large hybrid sturgeon is an important commercial fish that is distributed in the Northwest Atlantic Ocean, Sea of Japan, Yangtze, Pearl Rivers, and the Eastern Pacific Ocean. China is the largest country for sturgeon farming worldwide and its processing into caviar for export. The processing of caviar results in more than 700 tons of by-product generation every year. Sturgeon skin has been used to extract collagen (Luo et al., 2018; Abuine et al., 2019; Zhu et al., 2020). However, there have been no studies on photoprotective effects of sturgeon skin collagen peptides. Thus this study was performed to 1) optimize the hydrolysis conditions of sturgeon skin collagen peptides by DPPH radical scavenging activity, 2) investigate the effect of large hybrid sturgeon skin collagen hydrolysate against UVB irradiation-induced photodamage using mouse fibroblast L929 and zebrafish models, 3) screen the antioxidant peptide using 3D-QSAR models, then verifying the UVB photoprotect effect.

## 2 Materials and Methods

### 2.1 Materials and Reagents

The large hybrid sturgeon (*Huso Dauricus* ♀×*Acipenser Schrenckii* ♂) was provided by Fujian Longhuang Sturgeon Co., Ltd. (Fujian, China). L929 mouse fibroblasts were obtained from the Conservation Genetics CAS Kunming Cell Bank (Kunming, China). Flavourzyme and 1-diphenyl- 2-pycryl-hydrazyl (DPPH) were obtained from Beijing Solarbio Science & Technology Co., Ltd. (Beijing, China). Fetal bovine serum (FBS), RPMI1640, and phosphate-buffered saline (PBS) were purchased from Hyclone (Logan, UT, United States). The MDA assay kit was acquired from the Nanjing Jiancheng Institute of Biological Engineering (Nanjing, China). 3-(4,5-dimethylthiazol-2-yl)-5-(3-carboxymethoxyphenyl)-2-(4-sulfophenyl)-2H-tetrazolium (MTS) was obtained from Promega (Madison, WI, United States), phenazine methosulfate (PMS) and 2′,7′-dichlorofluorescein diacetate (DCFH-DA) was purchased from Sigma-Aldrich (St. Louis, MO, United States). The mouse pro-collagenⅠalpha ELISA kit was purchased from Abcam (Cambridge, MA, United States). The mouse MMP-1 ELISA kit was purchased from BBI Life Sciences Corporation (Shanghai, China). The mouse MMP-2 and MMP-3 ELISA kit were purchased from RayBiotech (Norcross, GA, United States).

### 2.2 Fish Skin Preparation

The skin was peeled off and thoroughly washed with distilled water, followed by cutting into small pieces (2 × 2 cm^2^). To remove non-collagenous proteins, fish skin was stirred in 10 volumes (v/w) of 0.1 M NaOH for 24 h at 4°C, following which it was defatted with 10% butyl alcohol with a solid/solvent ratio of 1:10 (w/v) for 24 h, and the solvent was changed every 12 h. Lactic acid was used during the skin swelling process. The fish skin was then stirred in 3% lactic acid for 2 h. The treated skin was washed with distilled water and homogenized. The prepared samples were stored at −20°C until use.

### 2.3 Preparation of Large Hybrid Sturgeon Skin Collagen Hydrolysates and Selection of Proteolytic Enzymes

Homogenized fish skin was mixed with double-distilled water (ddH_2_O) at a ratio of 1:10 v/w. The mixtures were adjusted to the required pH and heated in a water bath to the required temperature before seven proteases (acid proteinase, alkaline protease, pepsase, animal protease, flavourzyme, papain, and bromelain) were added in appropriate proportions based on enzymatic activity. At the end of the hydrolysis period, mixtures were heated in boiling water for 10 min to inactivate the proteases. Next, hydrolysates were centrifuged at 10,000 g (4°C) for 30 min. Supernatant was filtered using 0.45 μm syringe filters and stored at 4°C until use.

### 2.4 Single-Factor Experiment

The flavorzyme-treated hydrolysate was chosen as the best candidate, and four independent variables (enzyme concentration, pH, hydrolysis temperature, and hydrolysis time) were selected for the single-factor experiment. DPPH scavenging activity and degree of hydrolysis (DH) were measured to evaluate hydrolysates. The initial hydrolysis condition of flavorzyme was an enzyme concentration of 10,000 U/g, pH of 7.0, hydrolysis temperature of 50°C, hydrolysis time of 6.0 h, and water/material ratio of 15. The ranges of tested variables were as follows: enzyme concentrations of 2000, 4,000, 6,000, 8,000, and 10,000 U/g; pH of 6.0, 6.5, 7.0, 7.5, and 8.0; hydrolysis temperatures of 40, 45, 50, 55, and 60°C; and hydrolysis times of 0.5, 2.0, 4.0, 6.0, 8.0 and 10.0 h. While searching for the optimal condition of one variable, the values of the other variables were fixed.

### 2.5 DPPH Scavenging Activity Assay

DPPH radical scavenging activity was determined using a previously described method. In brief, the sample was mixed with DPPH solution (0.15 mM in 80% ethanol) in equal amounts. After incubating this mixture for 30 min, the absorbance was measured at 517 nm using a spectrophotometer (Tecan, Morrisville, NC, United States). The sample blank was handled in the same manner, except that 80% ethanol was used instead of the DPPH solution. Another control group was comprised of DPPH solution and ddH_2_O. Glutathione, which was positive control, was reacted with DPPH as same as samples. The calculation was as follows: (Eq. 1)

$$\text{DPPH scavenging activity}\left(\text{\%}\right)=\frac{\left(\text{Acontrol}-\text{Acontrol blank}\right)-\left(\text{Asamples}-\text{Asample blank}\right)}{\text{Acontrol}-\text{Acontrol blank}}\times 100\text{\%}$$ (1)

### 2.6 Determination of the Degree of Hydrolysis

The degree of hydrolysis can be used as an indicator of the percentage of cleaved peptide bonds. Amino nitrogen was determined according to the national standard 5,009.235–2016, using the Kjeldahl method to determine total nitrogen. The calculation was as follows: (Eq. 2)

DH(%)= (amino nitrogen/total nitrogen)×100% (2)

### 2.7 Response Surface Methodology

Based on preliminary multivariable screening, the chosen levels for three independent variables (hydrolysis temperature, hydrolysis enzyme concentration, and pH) were designed using a Box-Behnken design (BBD) of RSM. DPPH scavenging activity was selected as the measure of the combination of the independent variables. Design-Expert 11 software was used to develop the statistical design, RSM modeling, and data analysis. The independent variables and their code-variable levels are listed in Table 1. The responses obtained from each set of experimental designs were analyzed using multiple regressions to fit the quadratic polynomial model.

**TABLE 1:** Factors and level of response surface analysis.

Coding Levels	Hydrolysis Temperature (X1)	Factors	pH (X3)
		Hydrolysis Enzyme Concentration (X2)	
-1	35	6,000	6
0	40	8,000	6.5
1	45	10,000	7

According to Design Expert software, an analysis of variance table was generated, and the effect and regression coefficients of the linear, quadratic, and interaction terms were determined. *p*-values greater than 0.05 indicated that model terms were not statistically significant. The regression coefficient was used to perform statistical calculations, and the generated 3D surface was determined using the fitted polynomial equation.

### 2.8 Isolation and Characterization of Low-Molecular-Weight Sturgeon Skin Collagen Hydrolysates

The SSCH prepared by the above extraction process was separated by ultrafiltration using a 3 kDa ultrafiltration membrane, and the low-molecular-weight SSCH fraction (SSCH-L) was freeze-dried under vacuum. Gel permeation chromatography was used to determine the molecular weight distributions of the SSCH-L.

### 2.9 Cell Culture and Treatment

Murine L929 fibroblasts were grown in RPMI1640 supplemented with 10% FBS and 1% penicillin-streptomycin at 37°C and 5% CO_2_ in a cell culture incubator. Cells were seeded in 96-well plates, 24-well plates, or 9 cm culture dishes, and maintained overnight to allow adherent monolayer formation. Cells were starved by culturing in a medium containing 3% FBS for 12 h and then irradiated with the appropriate UVB dose using a UV spectral radiometer (VLX-3 W, Vilber Lourmat, France). Cells were then treated with different concentrations of SSCH-L (0–1 mg/ml) for 24 h.

### 2.10 Cell Viability Assay

Cell viability was evaluated using MTS with the CellTiter 96 AQ_ueous_ Non-Radioactive Cell Proliferation Assay kit (Promega, Madison, WI, United States), according to the manufacturer’s instructions. Briefly, L929 fibroblasts were seeded in 96-well plates at a concentration of 1 × 10^5^ cells/mL and allowed to attach overnight. After serum starvation by culturing in medium containing 3% FBS for 12 h, plates were washed thrice with PBS. Cells were exposed to 40 mJ/cm^2^ UVB irradiation in 20 μL of PBS and immediately incubated with different concentrations of SSCH-L for 24 h. 100 μL of MTS/PMS solution with 3% RPMI (MTS/PMS: 3% RPMI = 1:5) was added to cells and incubated for 40 min at 37°C and 5% CO_2._ The absorbance at 490 nm was measured using a microplate reader (Tecan, Morrisville, NC, United States). At least six independent experiments were conducted.

### 2.11 Scratch Wound Assay

The effect of SSCH-L on the mobility of L929 fibroblasts was assessed using a scratch wound assay, which measures spreading and migration capabilities of cells on the surface. The cell suspension (70 μL, 3 × 10^5^ cells/mL) was seeded into a plate containing a wound healing plug. After 24 h of culturing, sterile forceps were used to pull out the plug, and 2 ml of medium containing SSCH-L was added to the plate. The experimental groups were as follows: RPMI1640 medium with 10% FBS; RPMI1640 medium with 0.5% FBS; RPMI1640 medium with 0.5% FBS and different concentration of SSCH-L (0.25 mg/ml-1 mg/ml). The PH0 model was used to observe cell migration status at 0, 24, and 48 h after SSCH-L incubation under a light microscope (DMI8, Leica, Wetzlar, German).

### 2.12 Determination of Intracellular Reactive Oxygen Species Generation

2,7-diacetyl dichlorofluorescein diacetate (DCFH-DA) was used to detect intracellular ROS. L929 cells were seeded in 96-well black plates at a concentration of 1 × 10^5^ cells/mL. After 12 h of serum starvation, the cells were incubated with 25 μM DCFH-DA prepared in phenol red and FBS-free RPMI1640 medium for 30 min at 37°C in the dark. After two washes with pre-warmed PBS, the cells were exposed to 100 mJ/cm^2^ of UVB. Next, cells were incubated with different concentrations of SSCH-L (0.25, 0.5, and 1 mg/ml). After 1 h incubation, cells were investigated under a microplate reader measurement (excitation/emission: 485/525 nm).

### 2.13 Determination of Malondialdehyde Content

L929 fibroblast cells (2 × 10^5^ cells/mL) were seeded in 9 cm tissue-culture treated dishes overnight and starved for 12 h. The dishes were exposed to 40 mJ/cm^2^ UVB in 5 ml of PBS. After incubation for 24 h with different concentrations of SSCH-L (0.25, 0.5, and 1 mg/ml), cells were collected for measurement of MDA content with the MDA assay kit, according to the manufacturer’s instructions (Jiancheng Bioengineering Institute, Nanjing, China).

### 2.14 Total Ribonucleic Acid Extraction and Quantitative Polymerase Chain Reaction Amplification

L929 fibroblast cells were cultured in 24-well plates at a concentration of 2 × 10^5^ cells/mL overnight, then starved for 12 h. The cells were then exposed to 40 mJ/cm^2^ UVB in 125 μL of PBS. After washing with PBS thrice, cells were treated with different concentration of SSCH-L and incubated for 24 h. The total RNA sample was collected and extracted using an RNAprep Pure kit (Tiangen Biotech, Beijing, China) according to the manufacturer’s instructions. For cDNA synthesis, reverse transcription was performed with 1 μg total RNA using a PrimeScript RT Master Mix (Accurate Biotech, Hunan, China). Real-time qPCR was performed using a FastStart Universal SYBR Green Master kit on a LightCycler96 Real Time PCR System. The primers used for amplification of the target genes (*IL1β*, *IL-6*, *TNF-α*, *Cox-2*) and the reference gene (succinate dehydrogenase complex flavoprotein subunit A, *SDHA*) are listed in Supplementary Table S1. Relative quantification of target gene expression levels was performed using the 2^−ΔΔCt^ method (Livak and Schmittgen, 2001).

### 2.15 Determination of Pro-Collagen I and MMPs Content

L929 fibroblast cells (2 × 10^5^ cells/mL) were seeded into 6 cm culture dishes. Cells were exposed to 40 mJ/cm^2^ UVB and treated with different concentrations of SSCH-L for 24 h, which were collected for measurement of pro-collagen I content and MMP-1 using the Mouse Pro-Collagen I alpha 1 Simple Step ELISA Kit (Abcam, Cambridge, United States) and Mouse MMP-1 ELISA Kit (BBI life sciences corporation, Shanghai, China). The amount of total cellular protein was estimated using the PierceTM BCA Protein Assay Kit (Thermo Fisher Scientific, Waltham, United States). The cell culture supernatant was collected and concentrated 3 times in an ultrafilter tube prior to MMP-2 and MMP-3 measurement by ELISA. The ELISA kits for mouse MMP-2 and MMP-3 were purchased from RayBiotech (Norcross, GA, United States).

### 2.16 Zebrafish Embryo Culture, UVB Treatment, and Repair Experiments

Two days postfertilization (dpf) AB strain zebrafish were randomly divided into 6-well plates (30 embryos/well). Embryos were exposed to UVB generated by an ultraviolet light therapy instrument (KN-4006, Kernel Medical Equipment, Xuzhou, China). For UVB exposure, each group was exposed 3 times separated by 30 min intervals. Each exposure delivered 8,100 mJ/cm^2^ (9 mW/cm^2^, 15 min) of energy. After UVB exposure, all embryos were exposed to 3 ml water solution with or without different concentrations of SSCH-L (0.0625, 0.125, 0.25, 0.5, 1, 2, and 4 mg/ml).

For survival rates, embryos at 5 dpf were collected for observation. Embryos For protection experiments, ten zebrafish at 5 dpf were randomly selected from each group to observe their tails under a dissecting microscope (SZX7, Olympus, Tokyo, Japan). Images were taken and analyzed using an image processing software (NIS-Elements D 4.30.00) refer to the NIS-Elements BR (Basic Research) User’s Guide to calculate the area of the caudal fin of the larva. The caudal fin area was used to evaluate the effect of SSCH-L on the repair of UV irradiation-induced skin damage in zebrafish. The formula for SSCH-L for skin injury repair in zebrafish is as follows: (Eq. 3):

$$\text{Repair effect}\left(\%\right)=\frac{S_{\text{SSCH}}-S_{model\;control}}{S_{\text{model control}}}\times 100\%$$ (3)

### 2.17 Screening and Identification of the Potential Bioactive Peptides

The peptides were analyzed by online nano flow liquid chromatography tandem mass spectrometry performed on an EASY-nano LC 1000 system (Thermo Fisher Scientific, MA, United States) connected to a Q Exactive™ mass spectrometer (Thermo Fisher Scientific, MA, United States). Acclaim PepMap C18 (75 μm × 25 cm) as equilibrated with solvent A (A: 0.1% formic acid in water) and solvent B (B: 0.1% formic acid in ACN). 10 μL peptide sample was loaded and separated with 60 min-gradient at flow rate of 400 nL/min. The column temperature was 40°C. The electrospray voltage of 2 kV versus the inlet of the mass spectrometer was used. The peptides were eluted using the following gradient: 0–1 min, 1%–5% B; 1–35 min, 5%–30% B; 35–37 min, 30%–40% B; 37–38 min, 40%–90% B; 38–46.5 min, maintained 90% B; 46.5–47 min, 90%–1% B; 47–60 min, maintained 1% B.

The mass spectrometer was run under data dependent acquisition (DDA) mode, and automatically switched between MS and MS/MS mode. The survey of full scan MS spectra (m/z 200–1800) was acquired in the Orbitrap with 70,000 resolution. The automatic gain control (AGC) target at 3 × 10^6^ and the maximum injection time was 60 ms. Then the precursor ions were selected into collision cell for fragmentation by higher-energy collision dissociation (HCD), the collection energy was 27. The MS/MS resolution was set at 17,500, the AGC target at 5 × 10^4^, the maximum injection time was 50 ms, isolation window was 2.2 m/z, and dynamic exclusion was 20 s.

Tandem mass spectra were processed by PEAKS Studio version 10.6 (Bioinformatics Solutions Inc., Waterloo, Canada). PEAKS DB was set up to search the uniprot-Acipenser ruthenus (version 202,104, 22,006 entries) database assuming none as the digestion enzyme. PEAKS DB were searched with a fragment ion mass tolerance of 0.02 Da and a parent ion tolerance of 7 ppm. Oxidation (M), Deamidation (NQ) and Acetylation (Protein N-term) were specified as the variable modifications. The peptides with −10lgP ≥15 and the proteins with −10lgP ≥0 and containing at least 1 unique peptide were filtered.

The peptides with molecular weight less than 1 kD and -10lgP greater than 20 were screened out and were subsequently predicted with bioactive probability which was calculated using PeptideRanker server (Conway Institute of Biomolecular and Biomedical Research, University College Dublin, Ireland). Subsequently, 3D-QSAR pharmacophore model was generated and validated. The antioxidant 3D-QSAR model was constructed by Discover Studio 2019 client with the report antioxidative capacity database, which contains 20 samples (Supplementary Table S2) as training set and 10 samples as testing set (Supplementary Table S3) (Li et al., 2011; Liu et al., 2019). 3D-QSAR model was used to predict bioactive peptides from SSCH-L. The peptides with non-toxic and good solubility were finally screened by combining the results of ADMET property prediction and skin toxicity prediction in Discovery Studio module. The screened peptides were chemically synthesized by the solid phase procedure with the purity of 98%.

### 2.18 Comet Assay

L929 cells were exposed by 40 mJ/cm^2^ UVB and treated with DPFRHY for 24 h. The comet assay was performed using the reagents from the OxiSelect Comet assay kit (Cell Biolabs, San Diego, CA, United States) according to the manufacturer’s instructions. Comets were viewed using fluorescein microscope (DMI8, Leica, Wetzlar, German).

### 2.19 Hoechst 33342 Staining

The intensity of nuclear condensation was examined using the cell-permeable DNA dye Hoechst 33342. The probe (2 μg/ml) was added after 24 h of UVB exposure, and cells were incubated for 20 min at 37°C and washed twice with PBS. The cells were then visualized under a fluorescence microscope (DMI8, Leica, Wetzlar, German).

### 2.20 Western Blotting

Total proteins were extracted by RIPA lysis buffer with 1 mM PMSF. The amount of protein was estimated using the PierceTM BCA Protein Assay Kit (Thermo Fisher Scientific, Waltham, United States). Afterward, equal amounts of protein were collected and heated at 100°C for 5 min in a loading buffer. The proteins were electrophoresed on a 15% SDS-polyacrylamide gel and transferred to a polyvinylidene fluoride membrane (Pall Corporation, NY, United States) in a transfer buffer (25 mM Tris base, 250 mM glycine, 20% methanol). The membranes were blocked with 5% skim milk (BD Difco, NJ, United States) in PBS containing 0.05% Tween 20 buffer overnight at 4°C and incubated with following primary antibodies for 2 h at room temperature: p38 MAPK antibody (dilution 1:1,000; Cell Signaling Technology, Beverly, MA, United States, cat #9212), Phospho-p38 MAPK (Thr180/Tyr182) (D3F9) XP rabbit antibody (dilution 1:1,000; Cell Signaling Technology, cat #4511), p44/42 MAPK (Erk1/2) (137F5) rabbit antibody (dilution 1:1,000; Cell Signaling Technology, cat #4695), Phospho-p44/42 MAPK (Erk1/2) (Thr202/Tyr204) (D13.14.4E) XP rabbit antibody (dilution 1:1,000; Cell Signaling Technology, cat #4370), Recombinant Anti-JNK1 + JNK2 + JNK3 antibody (dilution 1:1,000, Abcam, Cambridge, MA, United States, cat #ab179461), Recombinant Anti-JNK1 + JNK2 + JNK3 (phospho T183+T183+T221) antibody (dilution 1:1,000, Abcam, cat #ab124956), and Recombinant Anti-SDHA antibody (dilution 1:2000, Abcam, cat #ab137040). Membranes were washed and incubated with horseradish peroxidase-conjugated goat anti-rabbit IgG secondary antibody for 1 h at room temperature. The antigen-antibody complex was detected by chemiluminescence using ECL detection reagent (Advansta, CA, United States) and analyzed using the ChemiDoc^®^ XRS+ Imaging System (Tanon-5200s, Tanon, Shanghai, China).

### 2.21 Statistical Analysis

Statistical analysis was performed using GraphPad Prism, and the significant difference compared to UVB model group was calculated via Bonferroni or Dunnett test. Prism reports results as non-significant (ns) at *p* > 0.05, significant (symbolized by “*“) at 0.01 < *p* ≤ 0.05, very significant (“**“) at 0.001 < *p* ≤ 0.01 and extremely significant (“***“) at *p* ≤ 0.001.

## 3 Results

### 3.1 Optimization of Protease Extraction of Sturgeon Skin Collagen Hydrolysates

#### 3.1.1 Selection of the Optimal Protease

**FIGURE 1:**
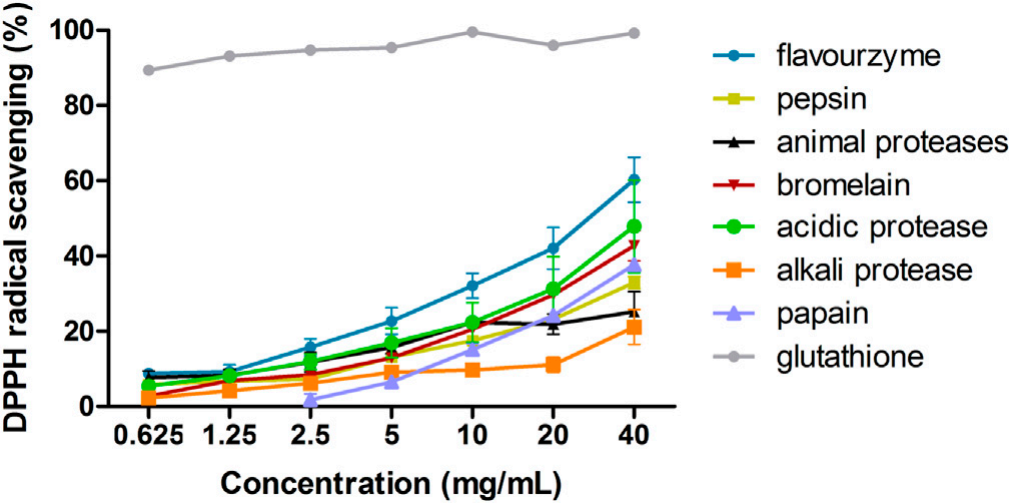
DPPH scavenging ability of seven proteases after hydrolysis.

We screened various proteases for the most efficient at producing hydrolysates. For each protease reaction, the same solid/liquid ratio, enzyme activity, and enzymolysis times were used with the optimal pH and temperature of each enzyme. All the hydrolysates showed a clear concentration dependency. The results are shown in Figure 1. As positive control, glutathione exhibited the strongest DPPH radical scavenging activity. Compared to other hydrolysates, the DPPH scavenging activity of flavourzyme enzymatic products was the highest. Therefore, flavourzyme was selected for subsequent modifications.

#### 3.1.2 Single-Factor Experiment

**FIGURE 2:**
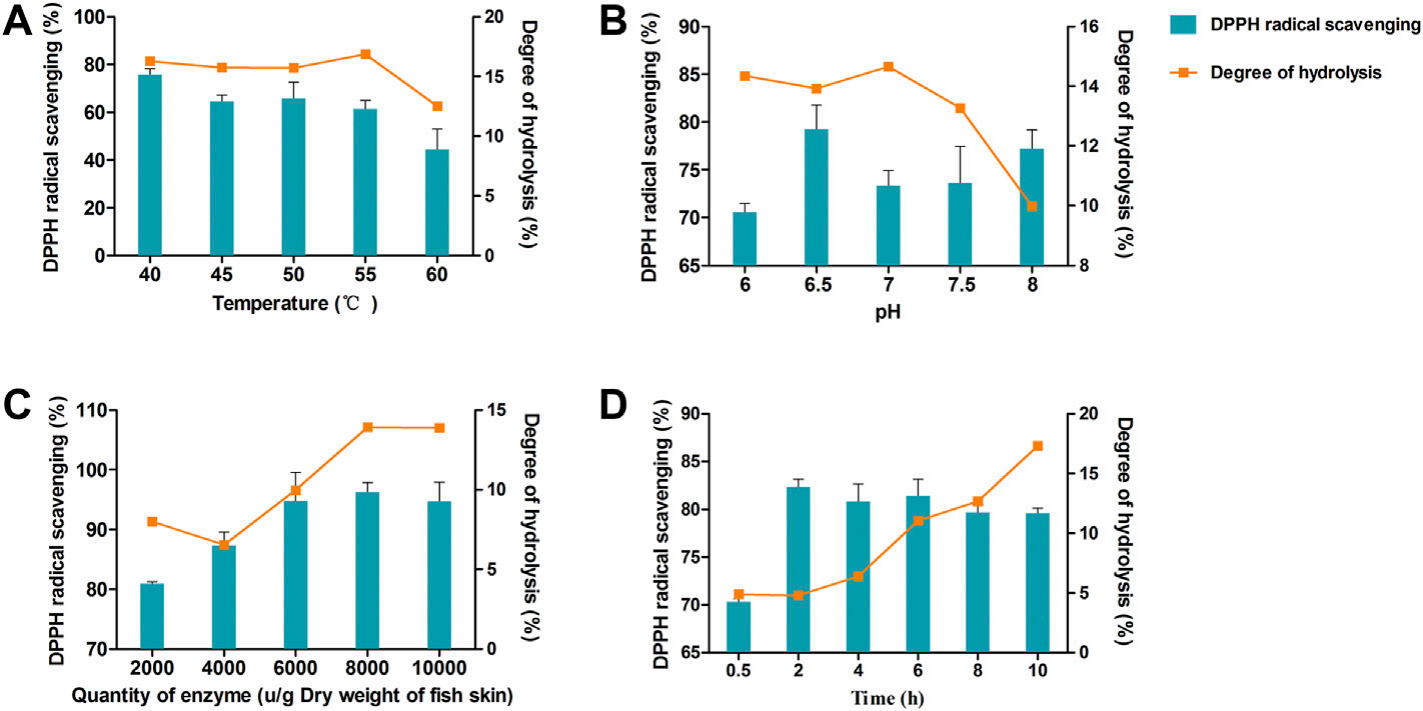
DPPH clearance rate and degree of hydrolysis (DH) vary with hydrolysis temperature **(A)**, pH **(B)**, enzyme dosage **(C)**, and time **(D)**.

Figure 2 illustrates the influence of the four variables (temperature, pH, enzyme concentration, and hydrolysis time) on rate of formation of DPPH radicals and the DH. As shown in Figure 2A, DPPH scavenging activity showed a downward trend from 40 to 60°C. DH demonstrated no significant difference between 40 and 55°C, but significantly decreased at 60°C.

The effects of pH 6–8 are shown in Figure 2B. DPPH scavenging activity reached a maximum at pH 6.5. The greatest DH of the enzymatic hydrolysate was between pH 6–7, and significantly declined after pH 7.

The concentration of enzyme was another important factor that could substantially influence DPPH scavenging activity and DH. As shown in Figure 2C, both DPPH radical rates and the DH increased with increasing enzyme concentration and reached a maximum at 8000 U/g dry weight of fish skin. When the concentration of enzyme increased beyond 8000 U/g, both DPPH radical rates and DH remained unchanged.

The hydrolysis time was evaluated at 0.5–10 h (Figure 2D). The DPPH radical rates reached a maximum at 2–6 h. However, DH continuously increased with time. Based on these results, we chose the following conditions as ideal for protease extraction of hydrolysates: temperature, 40°C; pH 6.5, enzyme concentration, 8000 U/g; and hydrolysis time, 6 h.

#### 3.1.3 Response Surface Methodology

##### 3.1.3.1 Model Fitting and Statistical Analysis

According to single-factor experiments, temperature, pH, and enzyme concentration were determined to be important factors for the optimized hydrolysis of SSCH. Based on the Box-Behnken statistical design, as shown in Table 2, a total of 17 experimental runs were required to investigate three parameters at three levels.

**TABLE 2:** Experimental design and results for optimization of extraction parameters by response surface methodology (RSM).

Run	pH	Temperature (°C)	Quantity of Enzyme (U/g)	DPPH Scavenging Rate (%)
1	6	45	8,000	95.1860
2	6	35	8,000	88.9700
3	6	40	10,000	95.5850
4	6.5	40	8,000	92.2474
5	6.5	35	10,000	89.2882
6	7	40	10,000	86.9541
7	7	40	6,000	91.1464
8	6	40	6,000	89.1549
9	6.5	40	8,000	93.1065
10	6.5	45	10,000	92.1057
11	6.5	35	6,000	94.6495
12	6.5	40	8,000	95.5645
13	6.5	45	6,000	87.2242
14	7	35	8,000	93.3113
15	6.5	40	8,000	91.3683
16	7	45	8,000	88.1525
17	6.5	40	8,000	92.6396

By applying multiple regression analysis to the experimental data, a second-order polynomial equation, which describes the correlation between the DPPH scavenging rate and the three variables, was obtained as follows: (Eq. 4):

$$Y=-453.49+\;108.23\text{A}+0.013\text{B}+7.61\text{C}-0.0026\text{AB}-1.144\text{AC }+0.00026\text{BC }-3.37A^{2}-3.58B^{2}\;-0.029C^{2}$$ (4)

where Y represents the DPPH scavenging rate, and A, B, and C represent the pH, quantity of enzyme, and temperature, respectively.

As shown in Table 3, the statistical significance of the model was determined by the *p*-value (*p* = 0.0193), indicating that the model was statistically significant. In addition, the F-value (lack of fit) of 0.9209 and the associated *p*-value of 0.5072 indicated an robust correlation between the predicted and experimental values. The determination coefficient (*R* ^2^ = 0.8772) and adjusted determination coefficient (R^2^_adj_ = 0.7079) were used to test the applicability of the model and indicated a satisfactory correlation of rate values predicted by the equation and those determined by experiment. Further, a low coefficient of variation (C.V.% = 1.69) indicated the reliability of the experimental values. These results suggested that the model will work well for the prediction of the DPPH scavenging rate of the hydrolysate.

**TABLE 3:** Analysis of variance for the fitted regression equation.

Source	Sum of squares	df	Mean square	F-value	*p*-value	Significance Level
Model	114.96	9	12.77	5.31	0.0193	significant
A-pH	0.1388	1	0.1388	0.0577	0.8171	
B-enzyme dosage	7.52	1	7.52	3.12	0.1205	
C-temperature	15.86	1	15.86	6.59	0.0372	
AB	28.21	1	28.21	11.72	0.0111	
AC	32.34	1	32.34	13.44	0.008	
BC	26.23	1	26.23	10.9	0.0131	
A^2^	2.99	1	2.99	1.24	0.3017	
B^2^	8.62	1	8.62	3.58	0.1002	
C^2^	2.28	1	2.28	0.9478	0.3627	
Residual	16.84	7	2.41			
Lack of Fit	6.88	3	2.29	0.9209	0.5072	not significant
Pure Error	9.96	4	2.49			
Cor Total	131.8	16				

R^2^ = =0.8772, adj.R^2^ = 0.7079, adeq.precision = =6.7413, and CV% = =1.69.

**FIGURE 3:**
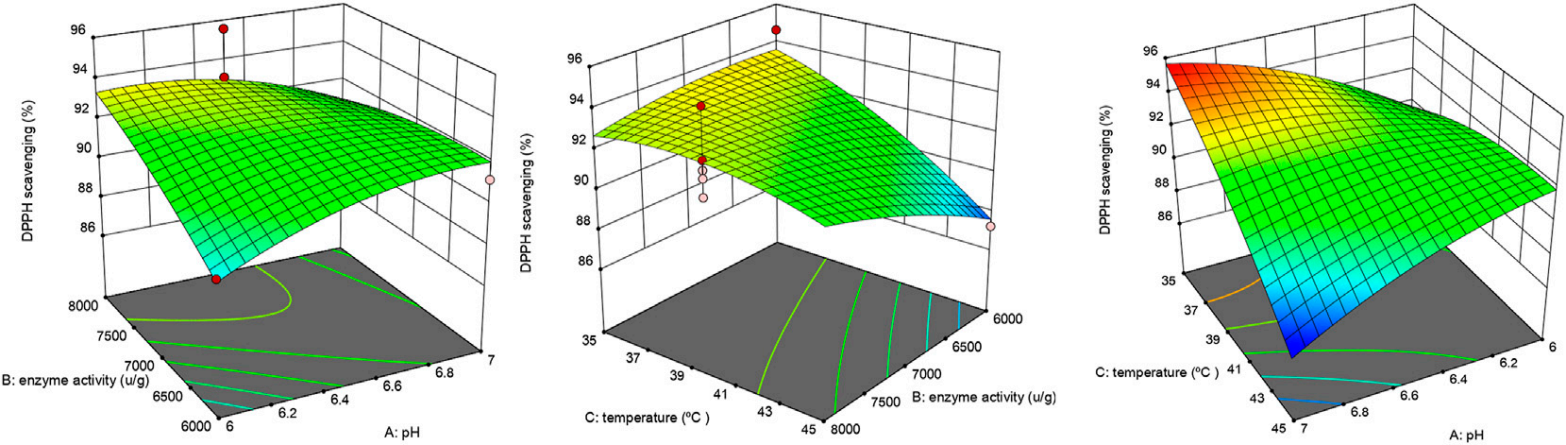
Interactive effect of extraction variables on DPPH scavenging activity.

The interaction of temperature and enzyme dosage showed a highly significant positive effect, and the interactive effect of AB and BC showed a significant positive influence. As shown in Figure 3, the interaction of 3D surface plots, the surface of BC was gentler than that of AB and AC, and the surface of AC was steeper than that of AB, suggesting that the interaction effect of AC > AB >BC.

##### 3.1.3.2 Validation of the Model

To determine the accuracy of the model equation, a verification experiment was carried out based on the optimal conditions: enzyme dosage 6,068.4 U/g, temperature 35.5°C, and pH 7. The mean DPPH scavenging rate (*n* = 3) was 95%, which closely correlated with the predicted value of 96%. This result validated the accuracy of the response model.

### 3.2 Protective Effect of Sturgeon Skin Collagen Hydrolysates-L on Ultraviolet B Induced L929 Cells Viability

The molecular weight distribution of the SSCH-L was measured, and the results are shown in the (Supplementary Table S4 and Figure S1). The average molecular weight of SSCH-L was 1996 g/mol, and the proportion below 2,550 g/mol was 71.3%.

**FIGURE 4:**
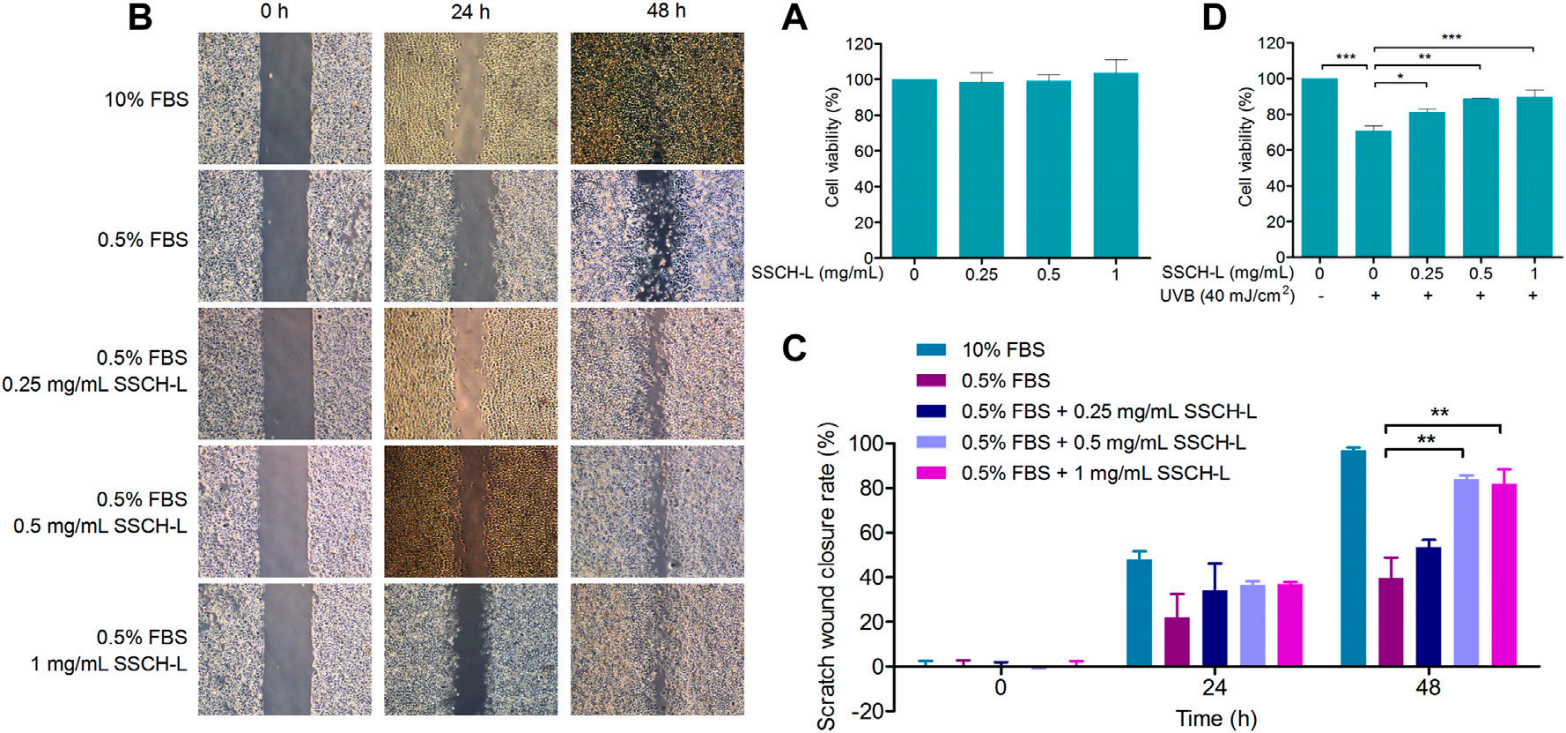
The effects of SSCH-L at different concentrations **(A)** on the viability and **(B)** the migration of L929 cells. **(C)** The wound closure rate was calculated at 0, 24, and 48 h after scratching. **(D)** The protective effects of SSCH-L on L929 cells damaged by UVB radiation. The error bars refer to standard deviations obtained from the triplicate sample analysis.

To determine the cytotoxicity of SSCH-L, L929 cells were treated with various concentrations of SSCH-L for 24 h. SSCH-L at concentrations below 1 mg/ml had no effect on cell viability relative to the control group (*p* > 0.05; Figure 4A). The migration rates of L929 cells treated with different concentrations of SSCH-L (0.25, 0.5, and 1 mg/ml) for 24 and 48 h are shown in Figures 4B,C. L929 cells gradually migrated to the scratch area over time, and the final amount of scratch area decreased with increasing SSCH-L concentration, showing a dose-dependent relationship. After 48 h, the best healing effect of SSCH-L was observed when the concentration of SSCH-L was 0.5 and 1 mg/ml. These results indicated that SSCH-L promoted the migration of L929 cells. As shown in Figure 4D, cell viability was reduced to 70% of the control value upon exposure to UVB. However, treating the cells with SSCH-L (0.5–1 mg/ml) significantly reversed the effect relative to UVB-treated cells.

### 3.3 Sturgeon Skin Collagen Hydrolysates-L Inhibits Intracellular Reactive Oxygen Species Generation and Lipid Peroxidation

**FIGURE 5:**
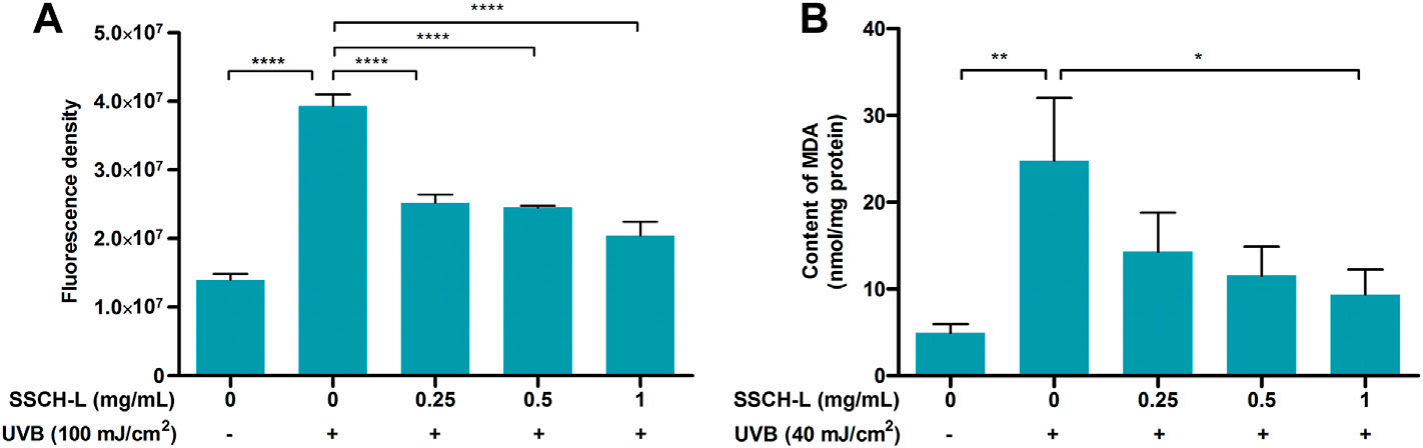
Effect of SSCH-L on intracellular reactive oxygen species (ROS) generation **(A)** and contents of malondialdehyde (MDA) **(B)** after L929 exposured to UVB.

Scavenging effects of UVB-induced ROS by SSCH-L were assessed via the fluorescent probe DCFH-DA (Figure 5A). Exposure to 100 mJ/cm^2^ UVB significantly promoted ROS production, which was more than tripled compared to that in the normal group. However, treatment of cells with SSCH-L reduced ROS production in a concentration-dependent manner. MDA content is an indicator of the lipid peroxidation rate and strength (Gil et al., 2002). As shown in Figure 5B, UVB irradiation significantly promoted lipid peroxidation, and SSCH-L reduced the MDA content induced by UVB irradiation in a concentration dependent manner. At a dosage of 1 mg/ml, the effect was statistically significant.

### 3.4 Sturgeon Skin Collagen Hydrolysates-L Alleviates Ultraviolet B-Induced Inflammation in L929 Cells

**FIGURE 6:**
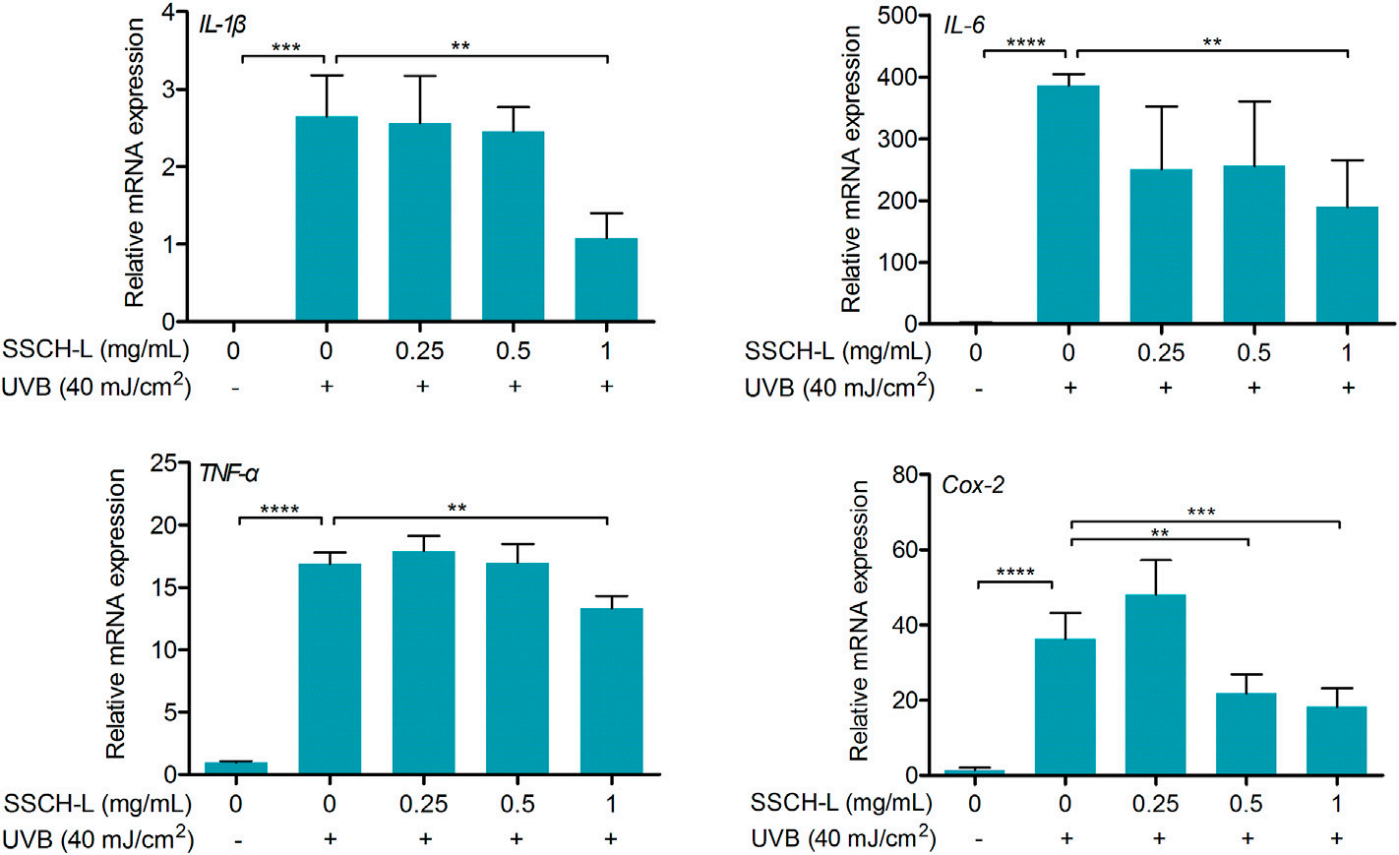
Effect of SSCH-L on the expression of pro-inflammation cytokines and *Cox-2* in the L929 cells exposed to 40 mJ/cm^2^ UVB.

UVB exposure stimulated the mRNA expression of pro-inflammatory genes, including *IL-1β*, *IL-6*, *TNF-α*, and *Cox-2* in L929 cells as compared to control cells (Figure 6). 1 mg/ml of SSCH-L reduced the expression levels of *IL-1β*, *IL-6*, *TNF-α*, and *Cox-2* by 2.5, 2.0, 1.3, and 2.0-fold, respectively.

### 3.5 Effect of Sturgeon Skin Collagen Hydrolysates-L Treatment on Collagen Synthesis and MMPs Expression

**FIGURE 7:**
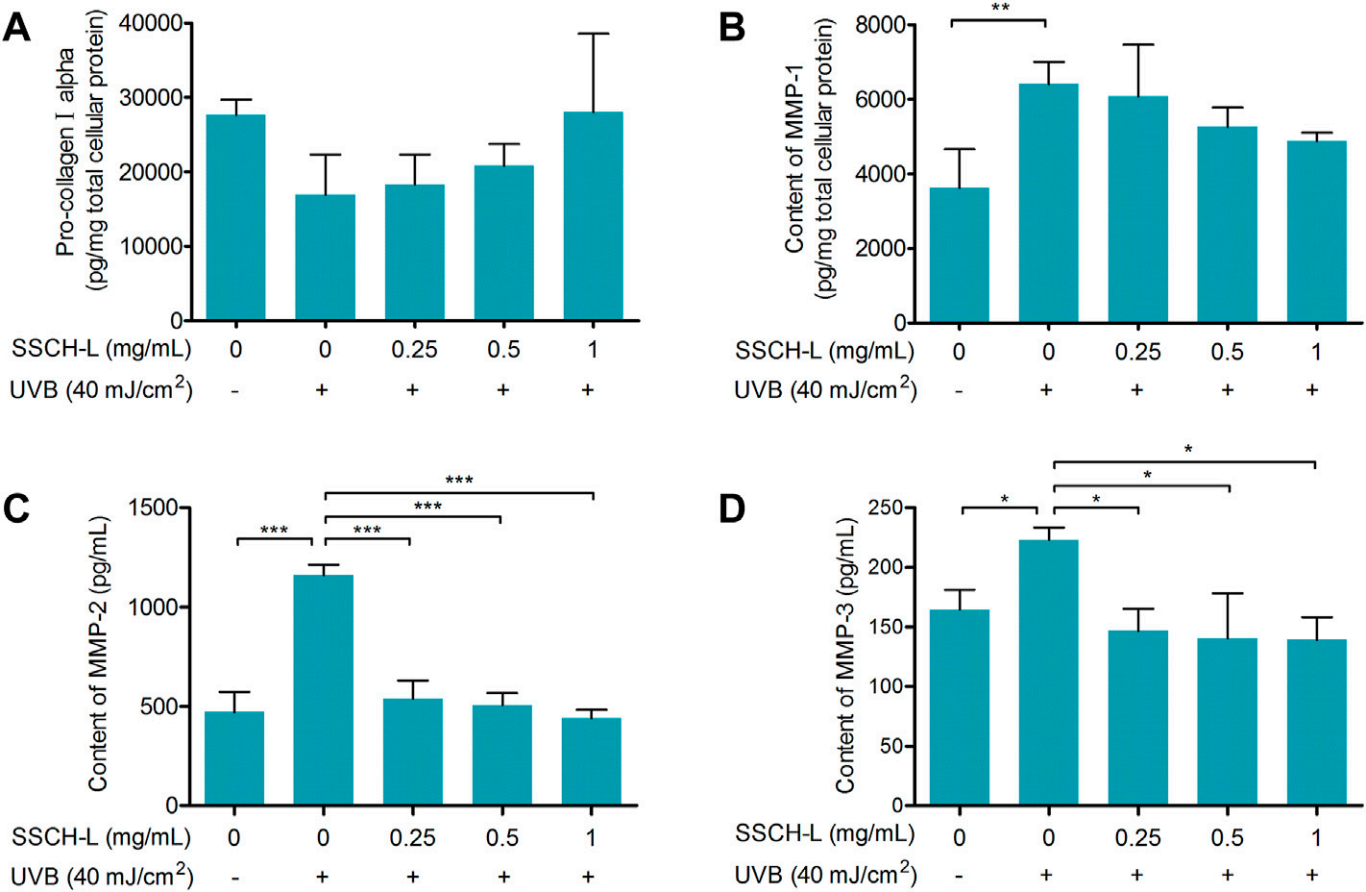
The protective effect of SSCH-L on procollagen degradation in UVB exposed L929. The protein levels of procollagen I alpha **(A)** and MMP-1 **(B)** in L929 cells and the levels of MMP-2 **(C)** and MMP-3 **(D)** in cell culture supernatant were detected by ELISA.

In this study, we assessed procollagen I alpha levels in UVB-irradiated L929 fibroblasts. 40 mJ/cm^2^ UVB reduced procollagen in L929 cells, and SSCH-L had a marginal counteractive effect on this (Figure 7A).

MMPs are the most prominent markers of collagen degradation, so we measured the expression of MMP-1, MMP-2, and MMP-3 to assess the effect of SSCH-L on UVB-induced cell damage. Figures 7B–D show that UVB irradiation significantly upregulated the protein content of MMPs. In contrast, the expression in the UVB-irradiated group with SSCH-L treatment was reduced in a concentration-dependent manner. MMP-1 was marginally decreased under SSCH-L-supplemented group compared to the UVB model group, while MMP-2 and MMP-3 were significantly decreased by all treatment groups.

### 3.6 Effects of Sturgeon Skin Collagen Hydrolysates-L Treatment on MAPK and AP-1 Pathways in L929 Cells

**FIGURE 8:**
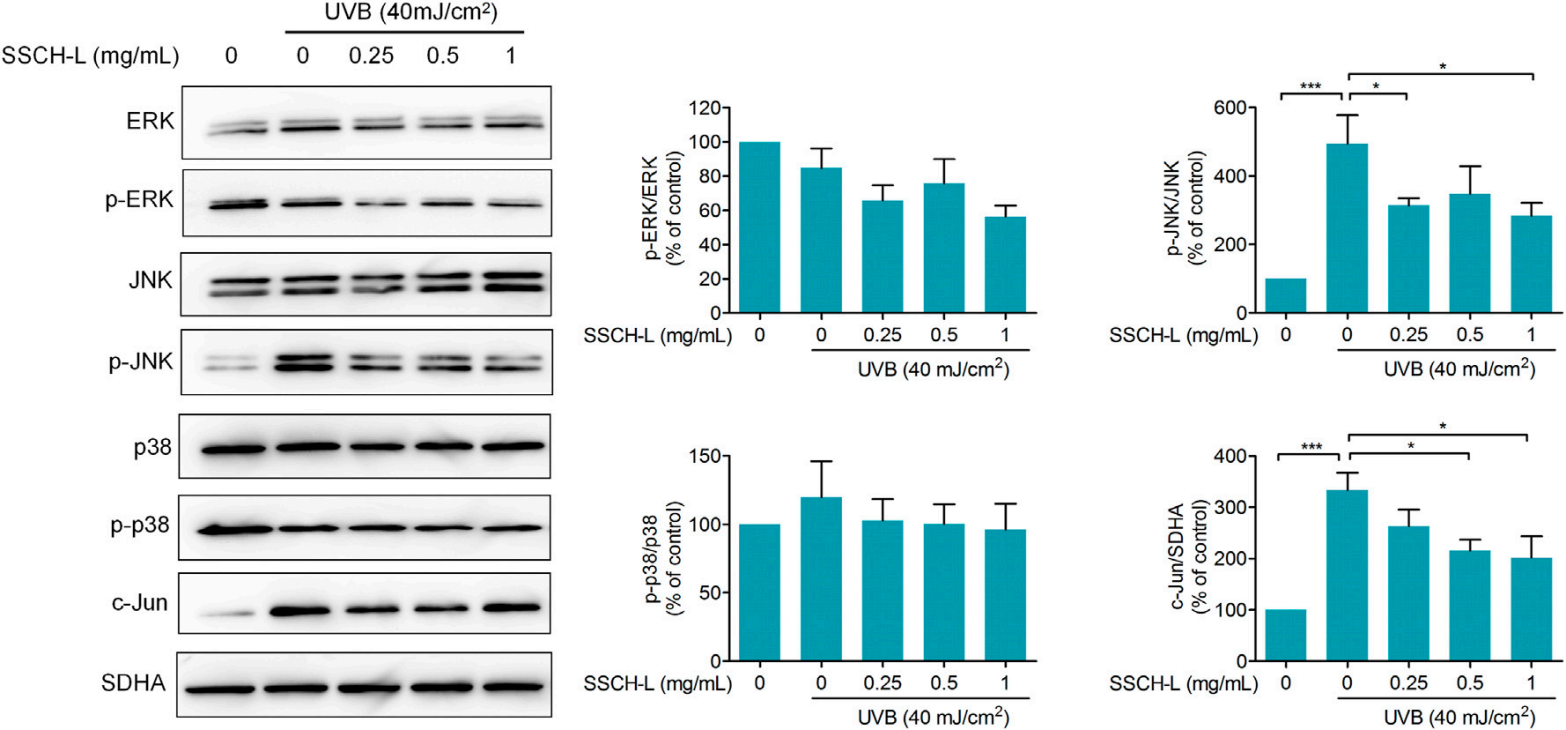
Effects of SSCH-L on MAPK and AP-1 signaling pathway in UVB-irradiated l929 cells. The intensities for phosphorylation levels of ERK, JNK, and p38 were measured by Western blotting. The results are shown as the mean ± SD of three independent experiments.

As MAPK and AP-1 signaling play vital role in regulating MMPs production, we evaluated treatment effects of SSCH-L on ERK, JNK, p38, and c-Jun expression. As shown in Figure 8, the downregulation of expression levels of phosphorylated JNK was observed after SSCH-L treatment. SSCH-L at the concentration of 0.5 and 1 mg/ml powerfully reduced c-Jun protein levels by 39.5, and 54.4%, respectively.

### 3.7 Repair of Caudal Fin Damage by Sturgeon Skin Collagen Hydrolysates-L in Ultraviolet B-Irradiated Zebrafish

Zebrafish have been widely used as an *in vivo* model to study protective effects of UV radiation. In this study, we first investigated the effect of SSCH-L on the growth of wild-type AB strain zebrafish (Supplementary Table S5). SSCH-L induced 16.7% of zebrafish deaths at concentrations of 0.5 mg/ml after UV irradiation. No significant abnormalities were observed at 0.0625–0.25 mg/ml SSCH-L; therefore, this was the maximum experimental concentration usable in the zebrafish UV damage model.

**FIGURE 9:**
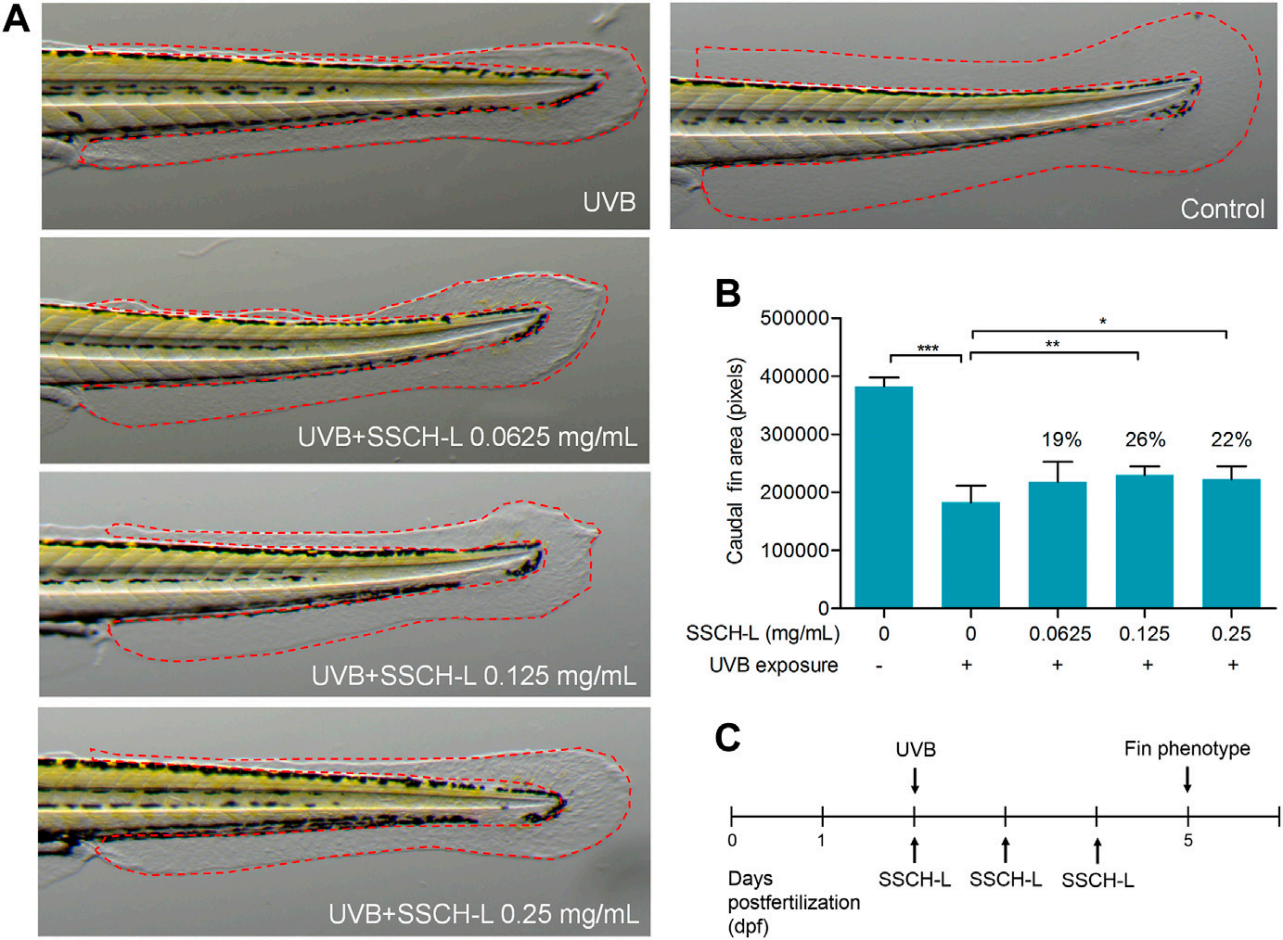
The potential of SSCH-L to repair skin damage in zebrafish exposed to UVB. **(A)**. UVB-induced malformed fin phenotypes can be attenuated by SSCH-L. **(B)**. Quantification of fin phenotypes. **(C)**. Schematic representation of the zebrafish experimental protocols performed in this study.

The potential of SSCH-L to repair skin damage in zebrafish exposed to UVB radiation is shown in Figure 9. Using a deconvolution microscope, we observed that the caudal fin of zebrafish displayed a rough and crinkled phenotype after exposure to UV radiation. Treatment of zebrafish with 0.0625, 0.125, or 0.25 mg/ml of SSCH-L resulted in a caudal fin area of 217,898 ± 10,877, 229,876 ± 4,668, and 222,453 ± 7,071 pixels, respectively. Compared with that of the control, the effects on skin damage repair were 19% (*p* > 0.05), 26% (*p* < 0.01), and 22% (*p* < 0.05), respectively. Collectively, SSCH-L showed a significant restorative effect on zebrafish skin damage.

### 3.8 Protective Effect of Sturgeon Skin Collagen Hydrolysates-L-Screened Peptides on Ultraviolet B Induced Damage

**FIGURE 10:**
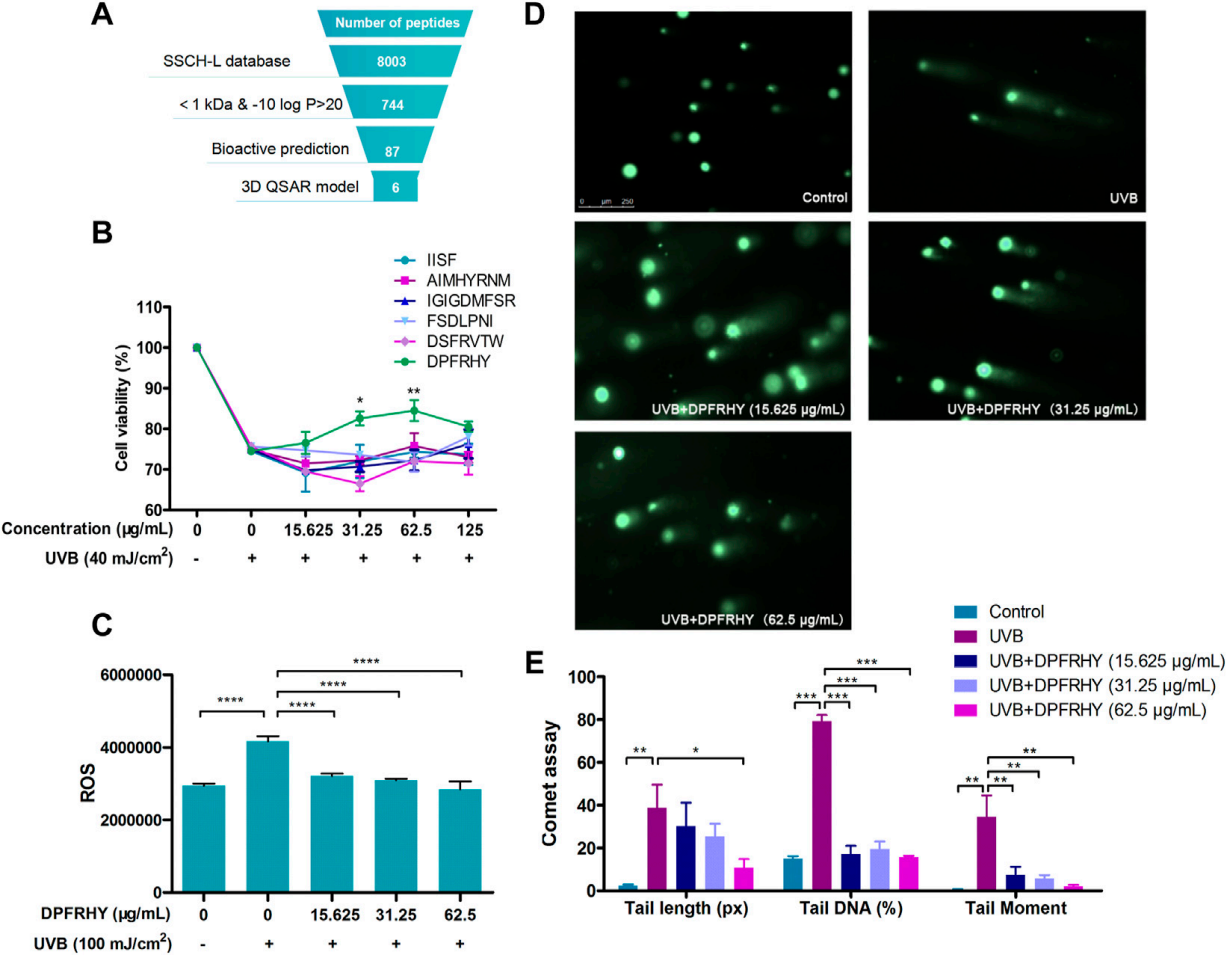
Screening and photo-protective effect of DPFRHY. **(A)**. Schematic representation of the virtual screening of antioxidant peptide from SSCH-L. **(B)**. The protective effects of 6 SSCH-L-screened peptides on L929 cells damaged by UVB radiation by cell viability assay. **(C)**. ROS scavenging effect of DPFRHY in UVB exposed L929 cell. **(D)**. Comet assay of L929 cells after UVB exposure and DPFRHY treatment. **(E)**. Quantification of comet scores of D by Cometscore 2.0 software.

Virtual screening has been widely used to explore natural bioactive compounds. In the present study, the virtual screening of antioxidant peptides proceeded based on 3D-QSAR modeling. The No.4 model possessed a higher match degree (*R* ^2^ = 0.739) than others (Supplementary Figure S2). Next, the No.4 model was chosen as queries against the SSCH-L database, in which 87 peptide sequences with bioactive probability and a molecular weight less than 1 kDa were selected. The screening procedure as show in Figure 10A, and six peptides were selected for further experiments.

The six SSCH-L-screened peptides were tested for their protective effects on UVB-induced damage. The sequences of the peptides were IGIGDMFSR, DSFRVTW, AIMHYRNM, FSDLPNI, IISF, and DPFRHY. As shown in Figure 10B, DPFRHY showed remarkable protective potency among the six compounds. DPFRHY at a concentration of 15.625–62.5 μg/ml significantly reduced the UVB-induced ROS content in L929 cells (Figure 10C). In the comet assay (Figure 10D), a long and obvious trailing phenomenon appeared in the UVB-irradiated L929 cells. When incubated with the DPFRHY peptide, the cell tail length was shorter than that in the UVB radiation group, and the tail DNA and tail moment were both significantly decreased with DPFRHY treatment. This indicated that the DPFRHY peptide could effectively repair the DNA damage caused by UVB.

### 3.9 DPFRHY Ameliorated Ultraviolet B-Induced L929 Apoptosis

**FIGURE 11:**
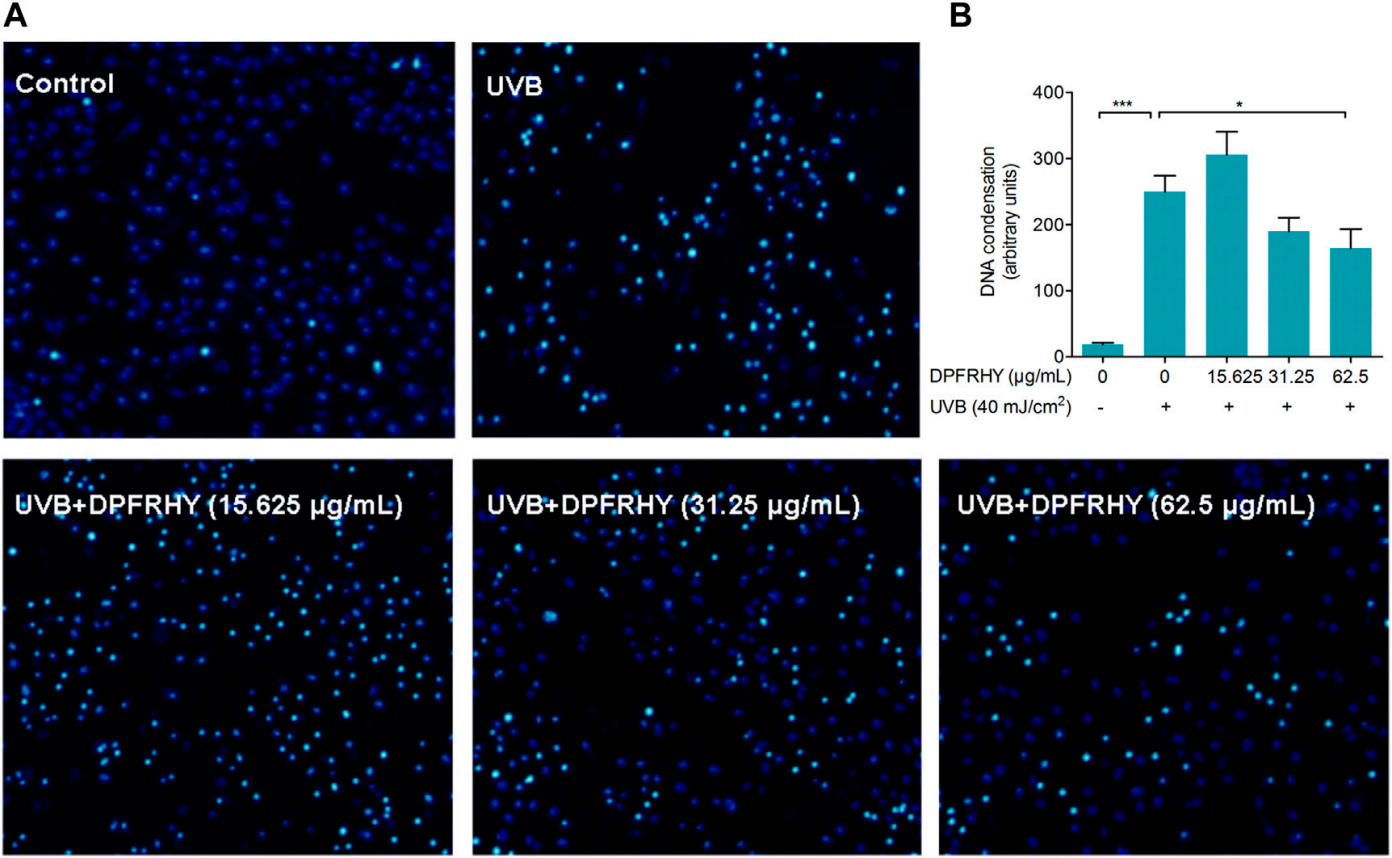
Evaluation of apoptosis protection in UVB-irradiated L929 fibroblasts treated with DPFRHY. **(A)** DNA condensation formation was observed under a fluorescence microscope following Hoechst 33342 staining. **(B)** The units statistics of DNA condensation in each field of microscope.

Chromatin condensation after UVB treatment was investigated by Hoechst assay (Figures 11A,B). The UVB irradiated group showed significant nuclear condensation (bright blue staining), suggesting these cells were undergoing the apoptotic process. After treatment with 62.5 μg/ml DPFRHY significantly reversed DNA condensation compared with the UVB control.

## 4 Discussion

The skin is the largest organ of the human body, playing a vital role as the first barrier in resisting outer environmental insults. However, the skin is fragile, and external environmental pollution can make it more vulnerable and can lead to various diseases. Among exogenous factors, UV irradiation is the main threat to the skin. UVB is a major cause of harmful biological effects and contributes to skin photoaging and inflammatory (Grether-Beck et al., 2014). Damage to the skin is directly or indirectly caused by over-activation of ROS. Previous studies have reported that over-production of ROS destroys the intracellular balance, causing oxidative stress, inflammation, and collagen breakdown (Wolfle et al., 2011). Thus, reducing oxidative stress would be a major strategy for protecting against UVB-induced photodamage.

DPPH radical-scavenging activity is commonly used as a response indicator in the extraction and optimization of antioxidant active components (Sonklin et al., 2018; Andres et al., 2020). Our study found that the sturgeon skin digest product of flavourzyme had the best DPPH activity. Several commercial proteolytic enzymes are used to hydrolyze proteins from fish byproducts (Zamora-Sillero et al., 2018). Flavourzyme possesses both endoprotease and exopeptidase activities, which have been suggested to be useful for the production of antioxidant peptides from fish protein, such as tilapia (Chuesiang and Sanguandeekul, 2015) and whitemouth croaker (Da Ros Zavareze et al., 2014). Previous studies have proven that low-molecular-weight peptides obtained from protein hydrolysis, such as collagen hydrolysate, can improve human skin functions (Kim et al., 2018). Meanwhile, it has been proven by many researches that an appropriately low molecular weight can exert a significant effect on the antioxidant activities of peptides (Zou et al., 2016). In addition, considering the application of SSCH in cosmetics and functional foods, low-molecule-peptides are more beneficial for intestinal and skin absorption. Based on these results, we obtained low-molecular-weight sturgeon skin peptides via ultrafiltration and examined their UVB-photoprotective effects.

ROS act as essential second messengers in the induction of various biological responses, such as the activation of NF-κB or AP-1, cytokine production, and the modulation of intracellular signaling pathways upon UVB exposure. Thus the intracellular ROS content and lipid peroxidation levels are important indicators to verify the photoprotective effects of active peptides against UVB radiation. Since the screened SSCH had good *in vitro* DPPH radical-scavenging activity, we hypothesized that SSCH-L could alleviate oxidative stress in UVB-exposed L929 cells. Our research revealed that levels of intracellular ROS generation and MDA content were significantly suppressed *in vitro* when L929 cells were treated with SSCH-L after UVB irradiation. To reduce the negative effect of UVB irradiation, many natural antioxidant peptides are used to improve the appearance of skin by decreasing ROS generation. For example, short gene-encoded peptide (OA-VI12) from *Odorrana andersonii* frog skin secretions (Yin et al., 2019), the new peptide OM-GL15 identified from the skin of the green odorous frog (*Odorrana margaretae*) (Zhang et al., 2021), and YGDEY from tilapia gelatin hydrolysates (Xiao et al., 2019) all have similar antioxidant functions in UVB-exposed skin cells or mouse models. Meanwhile, our findings demonstrated that UVB markedly induced the expression of inflammatory cytokines in L929 cells, which was significantly suppressed by treatment with SSCH-L. As there are multiple lines of evidence of cross-talk between inflammation and oxidants (Ballard-Croft et al., 2008), the antioxidant activities of SSCH-L might contribute to their anti-inflammatory effects.

The extracellular matrix (ECM), constituting over 70% of the skin, is composed mainly of collagen and elastin. UVB-induced imbalance between collagen degradation and synthesis strongly affects mechanical properties of ECM (Rittie and Fisher, 2015). MMPs are the main group of enzymes responsible for the degradation of collagen and other proteins in the ECM (Yamaba et al., 2016). Many studies have demonstrated that collagen or gelatin hydrolysate treatment can regulate the level of MMPs and consequently inhibit collagen degradation. (Fan et al., 2013) demonstrated that oral administration of jellyfish collagen hydrolysate has the ability to retain moisture and the histological appearance of mouse skin by inhibiting collagen degradation. (Liang et al., 2010) found that collagen extracts from Chum Salmon skin protect skin aging through the activation of the Smad signaling pathway and collagen degradation by decreasing MMP-1 expression. The increase of MMP-1 and MMP-9 activities induced by UVB irradiation was inhibited by tilapia fish skin gelatin hydrolysates in mouse embryonic fibroblasts (Ma et al., 2018). Oral administration of collagen tripeptide prevented UVB-induced MMP-3 and -13 activities as well as MMP-2 and -9 expressions in hairless mouse model (Pyun et al., 2012). Our results were consistent with these studies. Incubation with SSCH-L significantly downregulated the expression of MMP-1, MMP-2, and MMP-3, while the content of pro-collagen 1 alpha increased. Previous studies found that UVB-induced ROS production activates MAPK signaling pathway, and then stimulates MMPs gene transcription by the activation of AP-1 transcriptional factor (Lin et al., 2019). It has been suggested that activation of MAPK/AP-1 signaling plays a dominant role in inducing the release of MMPs. In order to understand the possible inhibitory mechanism of SSCH-L on MMPs expression, this study investigated the effects of SSCH-L on MAPK/AP-1 signaling pathway. The results demonstrated that SSCH-L significantly reduced the phosphorylation level of JNK and the expression of c-Jun protein. We could also observe a decreasing trend of p-ERK content under SSCH-L incubation. However p-ERK was not significantly upregulated after UVB irradiation. This might be related to the timing of our sampling. A study by (Chung et al., 2015) showed that the p-ERK content of mouse SP-1 keratinocytes was significantly upregulated at 15 h after UVB irradiation, followed by a decreasing trend. The sampling time of 24 h in our study may be the reason for the lack of significant difference in p-ERK between the UVB irradiated group and the control group.

The zebrafish model has become a popular animal model in pharmacological studies because of its small size, transparency, and genetic and physical behavior that is very similar to that of mammals. Zebrafish have been used to study the protective effects of natural extracts against UVB-induced fin damage (Su et al., 2020). Our work indicated that zebrafish fins in the UVB + SSCH-L groups were more likely to phenotypically return to normal than fins in the UVB only group. Fish collagen peptides to counter UVB damage of skin have already been applied to zebrafish models, such as the peptide derived from aquacultured flounder fish. The peptide reduce lipid peroxidation and ROS levels in UVB-irradiated zebrafish (Ko et al., 2014). The tilapia fish collagen peptide promotes skin wound healing in zebrafish as well as repair and proliferation of cells (Xiong et al., 2018).

In recent years, virtual high-throughput screening techniques have been widely used to identify new natural compounds. To screen antioxidant peptides from large food protein libraries, Tian et al. selected three antioxidant peptides with higher activity than glutathione *in vitro* by establishing a 3D-QSAR model (Tian et al., 2015). Yan et al. designed two antioxidant peptides according to the establishment of a 3D-QSAR model (Yan et al., 2020). Based on these studies, the 3D-QSAR model is a powerful tool to screen target peptides from huge natural compound libraries. Thus, we combined the oxygen free radical-scavenging pharmacophore 3D-QSAR model to screen antioxidant peptides with UVB-photoprotective activity. Our experimental results identified six antioxidant peptide hits, with four of the six peptides possessing antioxidant activity based on a ROS-scavenging assay *in vitro* (data not shown). However, only DPFRHY significantly increased the viability of cells after UVB radiation and had good anti-photodamage activity. This result indicates that antioxidants comprise only one of the many mechanisms of anti-photodamage effects. In other words, the photoprotective effect is the result of many mechanisms that function together.

UV radiation causes mutagenic DNA damage. Programmed cell death is an important mechanism to prevent the transformation of these DNA damages into skin cancer. Gary’s research indicates that apoptosis is the only known cell death mechanism induced by UVB irradiation in fibroblasts (Gary and Rochette, 2020). In the present study, we found DPFRHY reduced DNA damage caused by UVB radiation. Therefore the capacity of screened peptide DPFRHY to alleviate UVB-induced apoptosis in L929 was explored in depth. The DNA condensation was significantly reversed by DPFRHY, which suggest that DPFRHY protects fibroblasts against UVB damage by alleviating cellular apoptosis.

In this work, the skins of large hybrid sturgeon were used to prepare potential antioxidant peptides SSCH. We revealed that low-molecular-weight peptide SSCH-L has a protective effect against UVB induced photodamage, both *in vitro* using L929 cells, and *in vivo* using zebrafish models. The protective effect was mediated through antioxidant and anti-inflammatory activities and the inhibition of collagen degradation. One novel potential UVB photoprotective peptide (DPFRHY) with ROS scavenging activity, DNA damage protective activity and apoptosis inhibition activity was obtained by QSAR modeling prediction. This study should help to enlarge our understanding of the protective activity of antioxidant peptide on skin and should prompt the evaluation of DPFRHY as a potential protective agent to treat skin damage caused by UVB in pharmaceutical and cosmeceutical industries.

## Data Availability

The raw data supporting the conclusions of this article will be made available by the authors, without undue reservation.

## Abbreviations

dpf, days post-fertilization; ELISA, Enzyme-linked immunosorbent assay; FBS, fetal bovine serum; DCFH-DA, 2,7-dichlorodihydrofluorescein diacetate; MDA, malondialdehyde; MTS, 3-(4,5-dimethylthiazol-2-yl)-5-(3-carboxymethoxyphenyl)-2-(4-sulfophenyl)-2H-tetrazolium; PBS, phosphate-buffered saline; qPCR, quantitative PCR; ROS, reactive oxygen species; SDHA, succinate dehydrogenase complex flavoprotein subunit A; UV, ultraviolet; UVB, ultraviolet B; SSCH, sturgen skin collagen hydrolysates; DPPH, 1-diphenyl- 2-pycryl-hydrazyl; RSM, Response surface methodology.
