# Supplementary material for: Effects of Collagen Hydrolysate From Large Hybrid Sturgeon on Mitigating Ultraviolet B-Induced Photodamage
Source: Front Bioeng Biotechnol. 2022 Jun 27;10:908033. doi: 10.3389/fbioe.2022.908033 (PMC9271680; doi:10.3389/fbioe.2022.908033)
Supplement: Supplementary file 1 [file DataSheet1.docx]

Supplementary Material

**Table S1 Primers used in qPCR**

|  | **Forward primer (5’-3’)** | **Reverse primer (5’-3’)** |
| --- | --- | --- |
| *SDHA* | GGAACACTCCAAAAACAGACCT | CCACCACTGGGTATTGAGTAGAA |
| *TNF-α* | ACAAGGCTGCCCCGACTAC | TGGAAGACTCCTCCCAGGTATATG |
| *IL-1β* | CAACTGTTCCTGAACTCAACTG | GAAGGAAAAGAAGGTGCTCATG |
| *IL-6* | CCCAATTTCCAATGCTCTCC | TCCACAAACTGATATGCTTAGG |
| *Cox-2* | CAGGGAATGAGTACTGGGTCTATT | CAGGGAATGAGTACTGGGTCTATT |

**Table S2 QSAR database**

| **NO** | **Sequence** | **Activity** |
| --- | --- | --- |
| 1 | MHIRL | 0.306 |
| 2 | IANNEADAISLDGGQVFEAG | 0.43 |
| 3 | RWV | 0.51 |
| 4 | YVEEL | 0.799 |
| 5 | PWN | 0.906 |
| 6 | PIAAEVYEHTEGSTTSY | 1.68 |
| 7 | RWY | 2.762 |
| 8 | IAAEVYEHTEGSTTSY | 2.39 |
| 9 | WYSLAMAASDI | 2.621 |
| 10 | YAEERYPIL | 3.8 |
| 11 | GWNIP | 6.19 |
| 12 | WYSLAM | 7 |
| 13 | AGWNIP | 8.55 |
| 14 | GWNI | 13.9 |
| 15 | WNIP | 15.47 |
| 16 | LGFEY | 9.79 |
| 17 | VIPMGL | 2.89 |
| 18 | LGFEYY | 9.79 |
| 19 | LSKAQSDFG | -1.4 |
| 20 | AGWNIPIGT | 5.25 |

**Table S3 Testing database**

| **No** | **Sequence** | **Activity** |
| --- | --- | --- |
| 1 | PWM | 0.446 |
| 2 | PWY | 2.762 |
| 3 | LWH | 0.103 |
| 4 | LGFEY | 9.79 |
| 5 | WYSL | 4.52 |
| 6 | PIAAEVYEHTEGSTTSY | 1.68 |
| 7 | VIPMGL | 2.89 |
| 8 | IANNEADAISLDGGQVFEAG | 0.43 |
| 9 | IEWEGIESGSVEQA | 0.73 |
| 10 | LGFEYY | 7.25 |

**Table S4 Molecular weight distribution of SSCH-L**

|  | **Testing items** | **Results** |
| --- | --- | --- |
| **Molecular weight** | Mw/Mn | 1.647 |
|  | Mz/Mn | 2.180 |
|  | Mn | 1212 |
|  | Mp | 2532 |
|  | Mw | 1996 |
|  | Mz | 2643 |
| **Molecular weight distribution（%）** | 250.0-380.0 g/mol | 7.7 |
|  | 380.0 - 1400.0 g/mol | 25.0 |
|  | 1400.0 - 1630.0 g/mol | 18.6 |
|  | 1630.0-2550.0 g/mol | 20.0 |
|  | 2550.0-4382.0 g/mol | 28.7 |

**Table S5** **Effects of SSCH-L on the growth of wild-type AB strain zebrafish**

|  | **SSCH**  **Concentration（mg/mL）** | **Death rate**  **（%）** | **Toxicological phenotyping** |
| --- | --- | --- | --- |
| **Control** | - | 0 | No significant abnormalities were observed |
| **UVB** | - | 0 | No significant abnormalities were observed |
| **SSCH-L + UVB** | 0.0625 | 0 | No significant abnormalities were observed |
|  | 0.125 | 0 | No significant abnormalities were observed |
|  | 0.25 | 0 | No significant abnormalities were observed- |
|  | 0.5 | 16.7 | - |
|  | 1 | 100 | - |
|  | 2 | 100 | - |
|  | 4 | 100 | - |


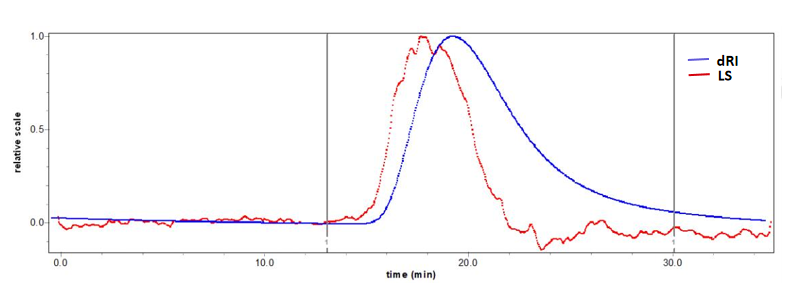


**Fig. S1 Gel permeation chromatography (GPC) spectra of SSCH-L**


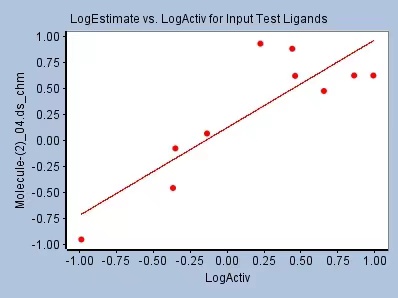


**Fig. S2 The correlation of antioxidate peptides model**
